# A promising leap in treating large granular lymphocytic leukemia: reflections on a Multicenter Phase II Study of thalidomide-based therapy

**DOI:** 10.1097/BS9.0000000000000239

**Published:** 2025-07-02

**Authors:** Xue Kong, Ken H. Young

**Affiliations:** 1 Duke University School of Medicine, Duke Cancer Center, Durham, NC 27710, USA

Large granular lymphocytic leukemia (LGLL) is a relatively uncommon malignancy of the blood system, marked by the clonal expansion of cytotoxic lymphocytes, particularly CD8+ T cells (T-LGLL), and in some cases, natural killer (NK) cells (NK-LGLL).^1^ Though rare, LGLL is clinically significant, representing approximately 2% to 6% of all chronic lymphoproliferative diseases, with a somewhat higher incidence in Asian populations.^2^ Clinically, patients often experience persistent cytopenias, most notably neutropenia and anemia, which may be accompanied by recurrent infections, splenomegaly, and a spectrum of autoimmune manifestations such as rheumatoid arthritis or Sjögren syndrome.^3^ Unlike acute leukemias, LGLL tends to follow a slow, indolent progression, but this belies the considerable impact it can have on patients’ daily functioning and long-term health.^4^

LGLL is closely associated with immune dysregulation, and considerable evidence points to aberrant signaling in the Janus kinase (JAK)/signal transducer and activator of transcription (STAT) pathway as a key driver of disease.^5^ Current frontline therapies therefore rely largely on immunosuppressive agents like methotrexate, cyclophosphamide, or cyclosporine.^6^ While these treatments can provide symptom relief and hematologic improvement in some patients, they are often limited by incomplete responses and high relapse rates, with complete remission achieved in only about half of cases.^7^ Furthermore, there is no universally accepted treatment protocol, and prospective, evidence-based studies in this field remain scarce, highlighting the urgent need for new therapeutic strategies.

Against this backdrop, the recently published phase II trial evaluating a novel triplet regimen, composed of thalidomide, prednisone, and methotrexate (TPM), offers promising results.^8^ This multicenter study enrolled 52 symptomatic LGLL patients between mid-2020 and 2022, including both newly diagnosed individuals and those who had previously undergone immunosuppressive treatment not involving thalidomide or methotrexate. By incorporating a clinically diverse population, the trial was able to assess the regimen’s potential across a broad range of real-world scenarios, including patients with severe anemia, concurrent autoimmune disorders, and transfusion dependence. Treatment involved a combination of thalidomide at a dose of 100 mg daily (continued for up to 2 years), prednisone on an alternate-day basis (tapered after 3 months), and methotrexate administered weekly. The study’s primary objective was to determine the rate of complete remission, while secondary endpoints included overall response rates, progression-free survival, duration of response, and safety. The results were compelling: three-quarters of patients achieved complete remission, and over 90% showed significant hematological or symptomatic improvement. These outcomes surpass historical benchmarks and position TPM as a potentially superior alternative to existing single-agent approaches. Moreover, therapeutic responses occurred relatively early—most patients showed improvement within 3 months, and complete responses were typically observed by 6 months. On top of this, the durability of response was notable, with a median progression-free survival of 40 months and a similar duration for sustained clinical benefit. The treatment not only alleviated symptoms but also led to meaningful hematologic normalization, with many patients achieving transfusion independence and improved neutrophil counts. Even those with initially severe cytopenias responded well, indicating the regimen’s effectiveness in high-risk subgroups.

Beyond clinical metrics, the study incorporated translational analyses to explore the underlying immunological changes induced by TPM therapy. Using multiplex cytokine profiling, researchers identified a number of pro-inflammatory mediators—such as interleukin (IL)-6, IL-8, and chemokine (C-C motif) ligand 3 (CCL3)—that were significantly elevated in LGLL patients before treatment. These markers decreased substantially following therapy, suggesting that the regimen exerts its effect, at least in part, by modulating inflammatory and immune signaling networks. This is particularly relevant given the central role of immune dysregulation in LGLL pathogenesis (Fig. 1).

**Figure 1.**
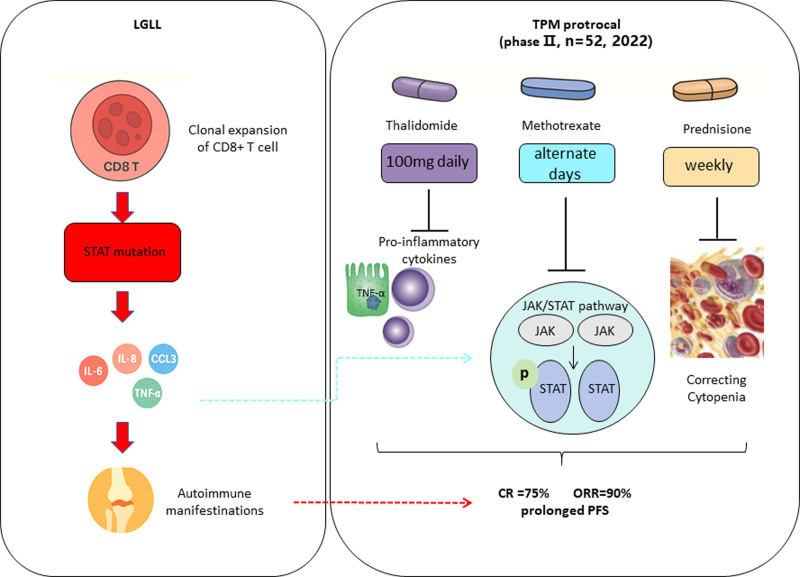
Proposed mechanism of action for the TPM protocol in LGLL. In LGLL, clonal expansion of CD8+ T lymphocytes often harbors STAT gene mutations, resulting in aberrant activation of inflammatory signaling cascades. This leads to elevated levels of pro-inflammatory cytokines such as IL-6, IL-8, CCL3, and TNF-α, ultimately contributing to autoimmune manifestations and cytopenias. The TPM protocol, consisting of thalidomide, prednisone, and methotrexate, targets these pathogenic pathways. Thalidomide reduces inflammation through inhibition of TNF-α and other cytokines; methotrexate suppresses clonal lymphocyte proliferation via inhibition of the JAK/STAT signaling pathway; prednisone rapidly mitigates cytopenias. Clinical outcomes of the multicenter phase II trial (n = 52, conducted in 2022) demonstrated a CR rate of 75%, an ORR of 90%, and prolonged PFS. CCL3 = chemokine (C-C motif) ligand 3, CR = complete remission, IL = interleukin-6, JAK = Janus kinase, LGLL = large granular lymphocytic leukemia, ORR = overall response rate, PFS = progression-free survival, STAT = signal transducer and activator of transcription, TPM = thalidomide, prednisone, and methotrexate, TNF-α = tumor necrosis factor-alpha.

Mechanistically, thalidomide is believed to inhibit tumor necrosis factor-alpha (TNF-α) and other inflammatory cytokines, while also enhancing T-cell–mediated immunity.^9^ Its immunomodulatory properties make it an ideal partner for methotrexate, which inhibits JAK/STAT signaling and suppresses clonal lymphocyte proliferation.^10^ Prednisone, meanwhile, serves a supportive role in quickly reducing lymphocyte counts and correcting cytopenias during the early treatment phase.^11^ Importantly, the study’s careful approach to steroid tapering helped mitigate the side effects commonly associated with long-term glucocorticoid use.

In terms of safety, the regimen proved generally well-tolerated. Peripheral neuropathy was the most frequent adverse effect, affecting about 1 in 4 patients, but was typically mild and manageable with dose adjustments or symptomatic care. Only a small number of patients experienced serious side effects, and no new safety concerns emerged with longer-term use of thalidomide beyond 2 years. This is especially noteworthy given that prolonged therapy is often necessary to maintain remission in indolent hematologic malignancies. Subgroup analysis further underscored the regimen’s broad applicability. Patients with T-LGLL, including both αβ and γδ T-cell receptor subtypes, responded similarly to those with NK-LGLL. The presence of STAT3 mutations, a known prognostic marker in LGLL, did not appear to influence treatment response, suggesting that the TPM regimen may overcome some of the resistance mechanisms associated with this genetic alteration.

Taken together, these findings suggest that TPM may represent a significant therapeutic advancement for LGLL. Its robust efficacy, favorable safety profile, and immunomodulatory mechanism offer clear advantages over traditional therapies. Nonetheless, it is important to interpret the results with caution. The study did not include a control arm, limiting the ability to draw direct comparisons to current standards of care. The relatively young median age of the study cohort (54 years) also means the results may not fully translate to older or more comorbid populations. Additionally, while the median follow-up of 29 months is adequate for early efficacy assessment, long-term outcomes such as late relapses or cumulative toxicities remain unknown. Moving forward, randomized phase III trials will be necessary to validate these findings and establish TPM as a new standard of care. Future studies might also explore optimal treatment duration, evaluate the potential role of cytokine profiles as predictive biomarkers, and investigate combination approaches with targeted agents or checkpoint inhibitors. Given the rarity of LGLL, such research will require coordinated multicenter efforts, but the potential benefits for patients are substantial.

In conclusion, this study provides the most compelling prospective evidence to date for the use of thalidomide-based therapy in LGLL. It opens the door to a new class of treatment strategies that go beyond simple immunosuppression, offering a more nuanced and potentially more effective approach to a challenging disease.

## References

[1] Lamy T Moignet A Loughran TP. LGL leukemia: from pathogenesis to treatment. Blood 2017;129(9):1082–1094. 28115367 10.1182/blood-2016-08-692590

[2] Marchand T Lamy T Loughran TP. A modern view of LGL leukemia. Blood 2024;144(18):1910–1923. 38848524 10.1182/blood.2023021790

[3] Lamy T Loughran TP. Clinical features of large granular lymphocyte leukemia. Semin Hematol 2003;40(3):185–195. 12876667 10.1016/s0037-1963(03)00133-1

[4] Drillet G Pastoret C Moignet A Lamy T Marchand T. Large granular lymphocyte leukemia: an indolent clonal proliferative disease associated with an array of various immunologic disorders. Rev Med Interne 2023;44(6):295–306. 37087371 10.1016/j.revmed.2023.03.014

[5] Teramo A Gattazzo C Passeri F. Intrinsic and extrinsic mechanisms contribute to maintain the JAK/STAT pathway aberrantly activated in T-type large granular lymphocyte leukemia. Blood 2013;121(19):3843–54, S1. 23515927 10.1182/blood-2012-07-441378

[6] Cheon H Dziewulska KH Moosic KB. Advances in the diagnosis and treatment of large granular lymphocytic leukemia. Curr Hematol Malig Rep 2020;15(2):103–112. 32062772 10.1007/s11899-020-00565-6 PMC7234906

[7] Magnano L Rivero A Matutes E. Large granular lymphocytic leukemia: current state of diagnosis, pathogenesis and treatment. Curr Oncol Rep 2022;24(5):633–644. 35212923 10.1007/s11912-021-01159-y

[8] Yu Y Li Y Cui R. Thalidomide-based regimen shows promising efficacy in large granular lymphocytic leukemia: a multicenter phase II study. Signal Transduct Target Ther 2025;10:85. 40069155 10.1038/s41392-025-02164-4 PMC11897152

[9] Quach H Mileshkin L Seymour JF. Predicting durable remissions following thalidomide therapy for relapsed myeloma. Leuk Lymphoma 2009;50(2):223–229. 19194832 10.1080/10428190802663213

[10] Thomas S Fisher KH Snowden JA Danson SJ Brown S Zeidler MP. Methotrexate is a JAK/STAT pathway inhibitor. PLoS One 2015;10(7):e0130078. 26131691 10.1371/journal.pone.0130078 PMC4489434

[11] Mithoowani S Gregory-Miller K Goy J. High-dose dexamethasone compared with prednisone for previously untreated primary immune thrombocytopenia: a systematic review and meta-analysis. Lancet Haematol 2016;3(10):e489–e496. 27658982 10.1016/S2352-3026(16)30109-0

